# Atorvastatin Attenuates Radiotherapy-Induced Intestinal Damage through Activation of Autophagy and Antioxidant Effects

**DOI:** 10.1155/2022/7957255

**Published:** 2022-08-31

**Authors:** Ming-Feng Wei, Ching-Hsueh Cheng, Shu-Yu Wen, Jui-Chueh Lin, Yu-Hsuan Chen, Chun-Wei Wang, Yi-Hsuan Lee, Sung-Hsin Kuo

**Affiliations:** ^1^Division of Radiation Oncology, Department of Oncology, National Taiwan University Hospital, National Taiwan University College of Medicine, Taipei, Taiwan; ^2^Graduate Institute of Oncology, National Taiwan University College of Medicine, Taipei, Taiwan; ^3^Cancer Research Center, National Taiwan University College of Medicine, Taipei, Taiwan; ^4^Division of Radiation Oncology, Department of Oncology, National Taiwan University Hospital HsinChu Branch, HsinChu, Taiwan; ^5^Department of Radiation Oncology, National Taiwan University Cancer Center, National Taiwan University College of Medicine, Taipei, Taiwan; ^6^Department of Pathology, National Taiwan University Hospital, National Taiwan University College of Medicine, Taipei, Taiwan; ^7^Medical Physics Program, College of Nuclear Science, National Tsing Hua University, Hsinchu, Taiwan

## Abstract

Abdominal or pelvic radiotherapy (RT) often results in small intestinal injury, such as apoptosis of epithelial cells and shortening of the villi. Atorvastatin, a 3-hydroxy-3-methylglutaryl coenzyme A reductase inhibitor, has many biological effects including cholesterol reduction, protection from cell damage, and autophagy activation. To reduce the extent of radiotherapy- (RT-) induced enteritis, we investigated the protective effects of atorvastatin against RT-induced damage of the intestinal tract. In this study, C57BL/6 mice were randomly distributed into the following groups (*n* = 8 per group): (1) control group: mice were fed water only, (2) atorvastatin group (Ator): mice were administered atorvastatin, (3) irradiation group (IR): mice received abdominal RT, (4) Ator+IR group: mice received abdominal RT following atorvastatin administration, and (5) Ator+IR+3-MA group: abdominal RT following atorvastatin and 3-methyladenine (an autophagy inhibitor) administration. Based on the assessment of modified Chiu's injury score and villus/crypt ratio, we found that atorvastatin administration significantly reduced intestinal mucosal damage induced by RT. Atorvastatin treatment reduced apoptosis (cleaved caspase-3 and cleaved poly (ADP-ribose) polymerase), DNA damage (*γ*H2AX and 53BP1), oxidative stress (OS, 4-hydroxynonenal), inflammatory molecules (phospho-NF-*κ*B p65 and TGF-*β*), fibrosis (collagen I and collagen III), barrier leakage (claudin-2 and fluorescein isothiocyanate-dextran), disintegrity (fatty acid-binding protein 2), and dysfunction (lipopolysaccharide) caused by RT in small intestinal tissue. In addition, atorvastatin upregulated the expression of autophagy-active molecules (LC3B), antioxidants (heme oxygenase 1 and thioredoxin 1), and tight junction proteins (occludin and zonula occludens 1). However, the biological functions of atorvastatin in decreasing RT-induced enteritis were reversed after the administration of 3-MA; the function of antioxidant molecules and activity of thioredoxin reductase were independent of autophagy activation. Our results indicate that atorvastatin can effectively relieve RT-induced enteritis through autophagy activation and associated biological functions, including maintaining integrity and function and decreasing apoptosis, DNA damage, inflammation, OS, and fibrosis. It also acts via its antioxidative capabilities.

## 1. Introduction

Most cancer patients receive radiotherapy (RT) as an important therapeutic modality in addition to surgery and chemotherapy [1]. However, radiation enteritis is a common sequela induced by RT [2]. It often occurs in patients with abdominal or pelvic cancer, including gastric, colorectal, hepatobiliary tract, pancreatic, cervical, and prostate cancer [2]. The causes for RT-induced enteritis include radiosensitivity of the intestinal cells (rapid cell turnover), apoptosis of the intestinal epithelium, shortening of villi, and intestinal injury caused by exposure to high-energy RT [2, 3]. Although most symptoms of acute enteritis, including vomiting, diarrhea, and bowel discomfort, can subside during or after RT, some patients may develop chronic enteritis and associated complications [4, 5]. Thereafter, the sequela of RT may affect their nutrient absorption and the overall quality of life [4, 5]. Therefore, the administration of RT-protective agents that maintain RT efficacy, while reducing RT-associated adverse effects, in such patients is warranted.

Statins are 3-hydroxy-3-methylgutaryl-coenzyme A (HMG-CoA) reductase inhibitors that act as rate-limiting enzymes and convert HMG-CoA to the cholesterol precursor mevalonate [6]. They are commonly prescribed for controlling hypercholesterolemia [6]. Additionally, statins have many beneficial effects, including antioxidant, anti-inflammatory, and antithrombotic effects [7–9]. Statins have also been reported to attenuate RT-induced fibrosis in head and neck cancers and RT-induced inflammation in various other cancers [10, 11]. Previous studies have shown that statins regulate tight junction formation and diminish the permeability of endothelial cells [12, 13]. RT may destroy certain tight junction proteins and inadvertently enhance the permeability of the intestinal epithelium, thus fostering intestinal damage [4, 5]. Therefore, exploring the administration of statins to rescue RT-induced impaired epithelial barrier function is recommended.

Physiologically, autophagy maintains cellular energy homeostasis and also regulates nutrient metabolism [14]. While carrying out their catabolic function, autophagosomes can degrade and recycle macromolecules as well as damaged organelles [14, 15]. Thus, they play an important role in the survival, differentiation, and development of cells [14, 15]. Additionally, autophagy can promote cell survival by attenuating RT-induced oxidative stress (OS) and cell damage [16, 17]. Previous studies have suggested that statins may protect different cells types from exogenous damage signals by activating autophagy-related signals [18–20]. Based on these findings, we hypothesized that statins could reduce RT-induced intestinal epithelial damage by activating cellular autophagy-related signals and via their antioxidant capabilities.

The present study explored whether atorvastatin administration could decrease RT-induced intestinal injury and cellular apoptosis in a mouse model. Additionally, the study also assessed whether atorvastatin administration enhanced the activation of autophagy-related signals. Thereby, anti-OS, anti-DNA damage, anti-inflammatory, and antifibrosis effects and effects on tight junction proteins, permeability, and integrity and function of the intestinal epithelium were also examined.

## 2. Materials and Methods

### 2.1. Animal Experiments

C57BL/6 male mice (6 weeks old) were purchased from the Animal Center of National Taiwan University College of Medicine. The Institutional Animal Care and Use Committee and the College of Public Health approved all the animal experimental procedures (IACUC approval number: 20150247). Mice were randomly distributed into the following groups (*n* = 8 per group)—(1) control group (Control): mice were fed only water and underwent laparotomy; (2) atorvastatin group (Ator): mice were administered atorvastatin (Sigma-Aldrich; 30 mg/kg/day) orally for five days; (3) irradiation exposure group (IR): mice received a dose of 5 Gy of abdominal irradiation for three days; (4) atorvastatin+irradiation group (Ator+IR) group: mice received a dose of 5 Gy of abdominal irradiation for three days, following oral atorvastatin administration (30 mg/kg/day) for five days; and (5) atorvastatin+irradiation+3-methyladenine group (Ator+IR+3-MA): mice received a dose of 5 Gy of abdominal irradiation for three days following atorvastatin administration (30 mg/kg/day) for five days accompanied by an intraperitoneal injection with 3-MA (autophagy inhibitor, 30 mg/kg/day) daily for five days (Supplementary Figure 1).

Blood was drawn for biochemical and hematologic analyses one day after the completion of the treatment regimen, and the mice were euthanized using carbon dioxide (CO_2_) inhalation. The jejunum was excised for histological and immunohistochemical analyses. The intestinal mucosa was harvested for quantitative real-time polymerase chain reaction (qRT-PCR) and western blot analyses.

### 2.2. Radiation Exposure Procedure

Mice were anesthetized via an intramuscular injection (1 mL/kg) with a mixture containing 25 mg/kg Zoletil 50 (Virbac Laboratories, Carros, France) and 2% xylazine (Bayer HealthCare Korea, Seoul, Korea), in a 2 : 1 ratio. 5 Gy radiation was administered to the abdomen using a Cs-137 irradiator (3 Gy/min). The remaining body parts were shielded with a lead block to prevent unwanted and untargeted irradiation (Supplementary Figure 2).

### 2.3. Blood Biochemistry and Hematologic Assays

Blood biochemistry assays were performed on mouse serum using the Roche Cobas c111 analyzer (Roche Diagnostics, Indianapolis, IN, USA). Levels of albumin (ALB), alanine aminotransferase (ALT), lactate dehydrogenase (LDH), and creatinine (CRE) were tested. For hematologic assays, blood was first collected into EDTA-containing tubes and then analyzed for hemoglobin (HGB) level, white blood cell count (WBC), and platelet count (PLT) using the IDEXX ProCyte Dx® Hematology Analyzer (IDEXX).

### 2.4. Histological Examination

The mice were dissected, and the abdominal wall was opened via a midline incision. Sections of jejunum—from the distal 10 cm of the pyloric sphincter to the proximal 10 cm of the cecum—were cut into optimally sized fragments (Supplementary Figure 2). The fragments were washed with ice-cold phosphate-buffered saline (PBS), fixed with 10% formaldehyde, and embedded in paraffin wax. Hematoxylin-eosin (H&E) staining was performed for morphometric assessment of the sections. In the current study, we used a modified Chiu's injury score to assess the degree of intestinal injury. These changes were evaluated based on changes in the villi and glands in the intestinal mucosa as seen in the H&E staining [21, 22]. In addition, the villus/crypt (V/C) ratio was determined by dividing the villus height by the crypt depth; a lower ratio indicated severe damage [23, 24]. Several investigators have also adopted Chiu's score or modified Chiu's injury score to assess mucosal injury of the small intestine from ischemia/reperfusion by histopathological evaluation [25, 26]. Histopathological changes were evaluated by investigators (Cheng CH and Wen SU) in a double-blinded fashion under supervision by a certified pathologist (Lee YH). Samples were graded based on the V/C ratio and modified Chiu's injury score [22].

The modified Chiu's injury score was classified as follows—grade 0: normal mucosal villi; grade 1: development of edema in Gruenhagen's space which is often accompanied by capillary congestion; grade 2: subepithelial space extension with moderate lifting of the epithelial layer from the lamina propria; grade 3: massive epithelial lifting along the villi sides, in addition to denuded apex; grade 4: denuded villi with exposed lamina propria, dilated capillaries, and an increased cellular infiltration within the lamina propria; and grade 5: disruption and disintegration of the lamina propria, presence of hemorrhage, and ulceration [21, 22, 25, 26]. At least five random fields were observed for each sample.

### 2.5. Western Blot Analysis

We harvested the mouse intestinal mucosa samples and extracted proteins using the Mammalian Protein Extraction Reagent (M-PER; Pierce). Equal volumes of protein extracts were separated using SDS-PAGE gels and transferred to PVDF membranes (Millipore). Membranes were blocked with 1X Tris-buffered saline containing 0.1% Tween and 5% nonfat dry milk. They were probed with primary antibodies of caspase-3 (#9662; Cell Signaling Technology, Danvers, MA), cleaved caspase-3 (#9661; Cell Signaling Technology), poly (ADP-ribose) polymerase (PARP) (#9532; Cell Signaling Technology), cleaved PARP (#94885; Cell Signaling Technology), microtubule-associated protein 1 light chain 3 beta (LC3B) (#3868; Cell Signaling Technology), sequestosome 1 (or p62) (ab56416; Abcam, Cambridge, UK), 4-hydroxynonenal (4-HNE) (ab46545; Abcam), heme oxygenase 1 (HO-1) (ab137749; Abcam), thioredoxin 1 (Trx1) (#2429; Cell Signaling Technology), glutathione peroxidase 4 (Gpx4) (MABF1969; Merck Millipore, Billerica, MA), H2AX (#7631; Cell Signaling Technology), γ;H2AX (#9718; Cell Signaling Technology), 53BP1 (ab172580; Abcam), NF-*κ*B p65 (#8242; Cell Signaling Technology), phospho (p)-NF-*κ*B p65 (#3033; Cell Signaling Technology), collagen I (sc-59772; Santa Cruz Biotechnology, Santa Cruz, CA, USA), collagen III (ab7778; Abcam), occludin (GTX85016; Genetex, Alton Pkwy Irvine, CA), zonula occludens (ZO)-1 (GTX108592; Genetex), claudin-2 (ab53032; Abcam), Ku80 (#2753; Cell Signaling Technology), glyceraldehyde-3-phosphate dehydrogenase (GAPDH) (#2118; Cell Signaling Technology), and *β*-actin (GTX11003; Genetex). Primary antibodies were detected using appropriate secondary IgG antibodies conjugated with horseradish peroxidase, and the specific reactive bands were visualized using the Western Lightning® Plus-ECL (PerkinElmer Inc.). Image Quant software (GE Healthcare) was used to quantify bands. All experiments were repeated three times.

### 2.6. Immunohistochemical (IHC) Analysis

Intestinal paraffin samples were cut into 4 *μ*m sections, deparaffinized, and stained with primary antibodies for cleaved caspase-3 (#9661; Cell Signaling Technology), cleaved PARP (#94885; Cell Signaling Technology), LC3B (#3868; Cell Signaling Technology), p62 (ab56416; Abcam), 4-HNE (ab46545; Abcam), HO-1 (ab137749; Abcam), Trx1 (#2429; Cell Signaling Technology), Gpx4 (MABF1969; Millipore), *γ*H2AX (#9718; Cell Signaling Technology), 53BP1 (ab172580; Abcam), p-NF-*κ*B p65 (ab86299; Abcam), collagen I (sc-59772; Santa Cruz), collagen III (ab7778; Abcam), occludin (GTX85016; Genetex), ZO-1 (GTX108592; Genetex), and claudin-2 (ab53032; Abcam). Images of the stained sections were acquired using the Olympus BX51 microscope with an Olympus DP72 camera and cellSens Standard 1.14 software (Olympus, Germany).

### 2.7. Quantitative Real-Time Polymerase Chain Reaction

We used the RNeasy Mini Kit (74104; Qiagen) to extract total RNA from the intestinal mucosa. The SuperScript III First-Strand Synthesis System was used for the reverse transcription-polymerase chain reaction (RT-PCR) (18080-051; Invitrogen). The mRNA expression levels of NF-*κ*B, TGF-*β*, collagen I, and collagen III were quantified using the Applied Biosystems 7900HT Fast Real-Time PCR System (Applied Biosystems) and normalized using GAPDH. The forward and reverse primer sequences for these genes were as follows: forward-AAG ACA AGG AGC AGG ACA TG and reverse-AGC AAC ATC TTC ACA TCC CC for NF-*κ*B; forward-CCT GAG TGG CTG TCT TTT GA and reverse-CGT GGA GTT TGT TAT CTT TGC TG for TGF-*β*; forward-CAT AAA GGG TCA TCG TGG CT and reverse-TTG AGT CCG TCT TTG CCA G for collagen I; forward-GAA GTC TCT GAA GCT GAT GGG and reverse-TTG CCT TGC GTG TTT GAT ATT C for collagen III; and forward-CAT GAG AAG TAT GAC AAC AGC CT and reverse-AGT CCT TCC ACG ATA CCA AAG T for GADPH. The experiment was repeated three times.

### 2.8. Assay for Thioredoxin Reductase (TrxR) Activity

We used a colorimetric TrxR assay kit (ab 83463; Abcam) to detect TrxR activity. Briefly, equal amounts of intestinal tissue were homogenized in cold TrxR assay buffer and centrifuged for 15 min at 10,000 × *g* at 4°C. Subsequently, the supernatant was stored on ice for further analysis. TrxR activity was assessed based on the conversion of 5,5-dithiobis (2-nitrobenzoic) acid to 5-thio-2-nitrobenzoic acid (TNB), catalyzed by TrxR. Absorbance of the yellow color was measured at 412 nm, with appropriate background control, using a spectrophotometer. TrxR activity was defined as mU/mg protein (1 U of TrxR activity was defined as 1.0 *μ*mol TNB formation per minute).

### 2.9. In Vivo Assessment of Intestinal Epithelial Permeability

We analyzed intestinal epithelial permeability by measuring the serum levels of 4 kDa fluorescein isothiocyanate- (FITC-) dextran (Sigma-Aldrich) in all the experimental groups. A day after the treatment regimen, a 20 mL/kg dose (or 25 mg/mL) of FITC-dextran was administered to each mouse in all experimental groups, by oral gavage, 3 h prior to blood collection. Next, blood samples in serum collection tubes were centrifuged at 10,000 × *g* for 10 min at room temperature to isolate the serum. Serum samples (diluted 1 : 3 with PBS) and the standards were added to 96-well plates to evaluate the intensity of FITC by spectrophotofluorometry (Wallac Victor; PerkinElmer Inc.) at 485 excitation/530 emission wavelengths. The concentration of FITC-dextran was then calculated using standard curves (range 0.0001–1 mg/mL).

### 2.10. In Vivo Assessment of Intestinal Barrier Integrity and Function

In all the experimental groups, we assessed intestinal epithelial integrity and intestinal barrier function by measuring the plasma levels of intestinal fatty acid-binding protein (i-FABP; FABP2) and lipopolysaccharide (LPS), respectively. Briefly, plasma was isolated from the blood one day after the treatment regimen ended. Plasma FABP2 and LPS levels were quantified using enzyme-linked immunosorbent assay (ELISA) kits (Cloud-Clone Corp., USA) according to the manufacturer's guidelines. Optical density (OD) was measured at 450 nm using a spectrophotometer (Wallac Victor; PerkinElmer Inc.). Plasma levels of FABP2 and LPS were expressed in ng/mL.

### 2.11. Statistical Analysis

The experimental data are expressed as mean ± standard error (SE). Statistical analyses were performed using *t* tests for multiple comparisons. Statistical significance was set at *P* < 0.05.

## 3. Results

### 3.1. Atorvastatin Administration Protects the Intestine from RT-Induced Injury

This study assessed the protective effect of atorvastatin against RT-induced intestinal epithelial damage by designing different treatment regimens in a murine model (Supplementary Figure 1). A schematic diagram of the experimental process is shown in Supplementary Figure 2. We studied if atorvastatin administration affects hemograms, liver function, and renal function. We also assessed WBC, HGB, PLT, ALB, ALT, LDH, and CRE levels in all the four groups. No significant differences were found in these parameters among the groups; however, the LDH level was lower in the Ator+IR group than in the IR group (Supplementary Table 1). These results implied that atorvastatin pretreatment did not impact RT-induced damage as determined by hemograms and liver or renal function. Moreover, atorvastatin administration did not aggravate the variation in blood homeostasis resulting from radiation exposure.

**Figure 1:**
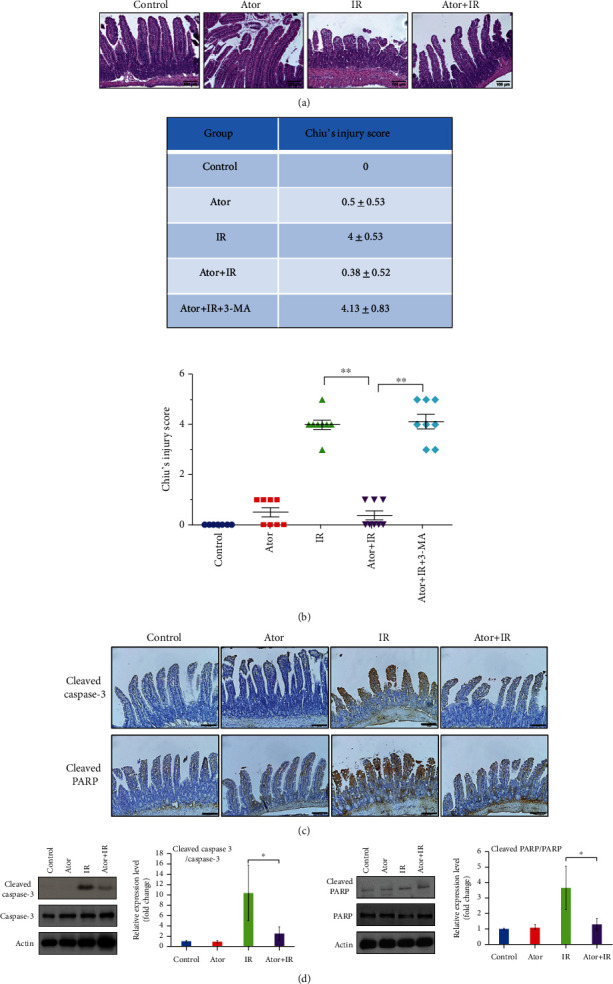
Role of atorvastatin in attenuating radiotherapy- (RT-) caused injury and apoptosis of intestinal epithelial cells. (a) Representative hematoxylin-eosin (H&E) staining images of jejunum tissue sections from four groups. Scale bar = 100 *μ*m. (b) Modified Chiu's injury score is presented for each group. Data are presented as mean ± standard error (SE) (*n* = 8). ^∗∗^*P* < 0.01. (c) Immunohistochemistry (IHC) analysis was used to assess the expression of cleaved caspase-3 and cleaved PARP in jejunum tissue sections. Scale bar = 100 *μ*m. (d) The protein expression levels of cleaved caspase-3 and cleaved PARP were analyzed by western blot analysis. The bar graph represents the relative ratios of the cleaved protein to the noncleaved protein. The data are presented as mean ± SE of three independent experiments. The relative ratio of the control group is arbitrarily presented as 1. ^∗^*P* < 0.05.

H&E staining of the jejunum tissue sections from four different groups (control, Ator, IR, and Ator+IR groups) is shown in Figure 1(a). The modified Chiu's injury parameters (Figure 1(b)) and the V/C ratio (Table 1) in the four groups were also assessed. In the control group, the microvilli of the small intestinal were structurally intact, and the modified Chiu's injury score was “0.” As compared with the Ator group (score: 0.5 ± 0.530), the IR group displayed morphological damage, including villi degeneration, shortening, and denuding. Lamina propria was exposed, and hemorrhage was observed in the jejunum mucosa (score: 4 ± 0.53) (Figure 1(b)). Administration of atorvastatin decreased RT-induced intestinal injury (score: 0.38 ± 0.52). As shown in Table 1, the V/C ratio was significantly lower in the IR group (1.41 ± 0.43) than in the control group (3.34 ± 0.96) and the Ator group (2.84 ± 1.04). The Ator+IR group (2.52 ± 0.89) also displayed a higher V/C ratio than that of the IR group. Thus, atorvastatin pretreatment attenuated the intestinal epithelial damage induced by RT.

The IHC staining and western blot results of the control, IR, and Ator groups revealed that the IR group had increased expression levels of cleaved caspase-3 and cleaved PARP when compared with control group. However, this effect was lowered by atorvastatin (Figures 1(c) and 1(d)). These results indicate that atorvastatin administration markedly reduced RT-induced apoptosis in the intestinal epithelial cells.

### 3.2. Autophagy Activation Stimulated by Atorvastatin after Radiation Enhances Its Radioprotective Effect on the Intestinal Tissue

**Figure 2:**
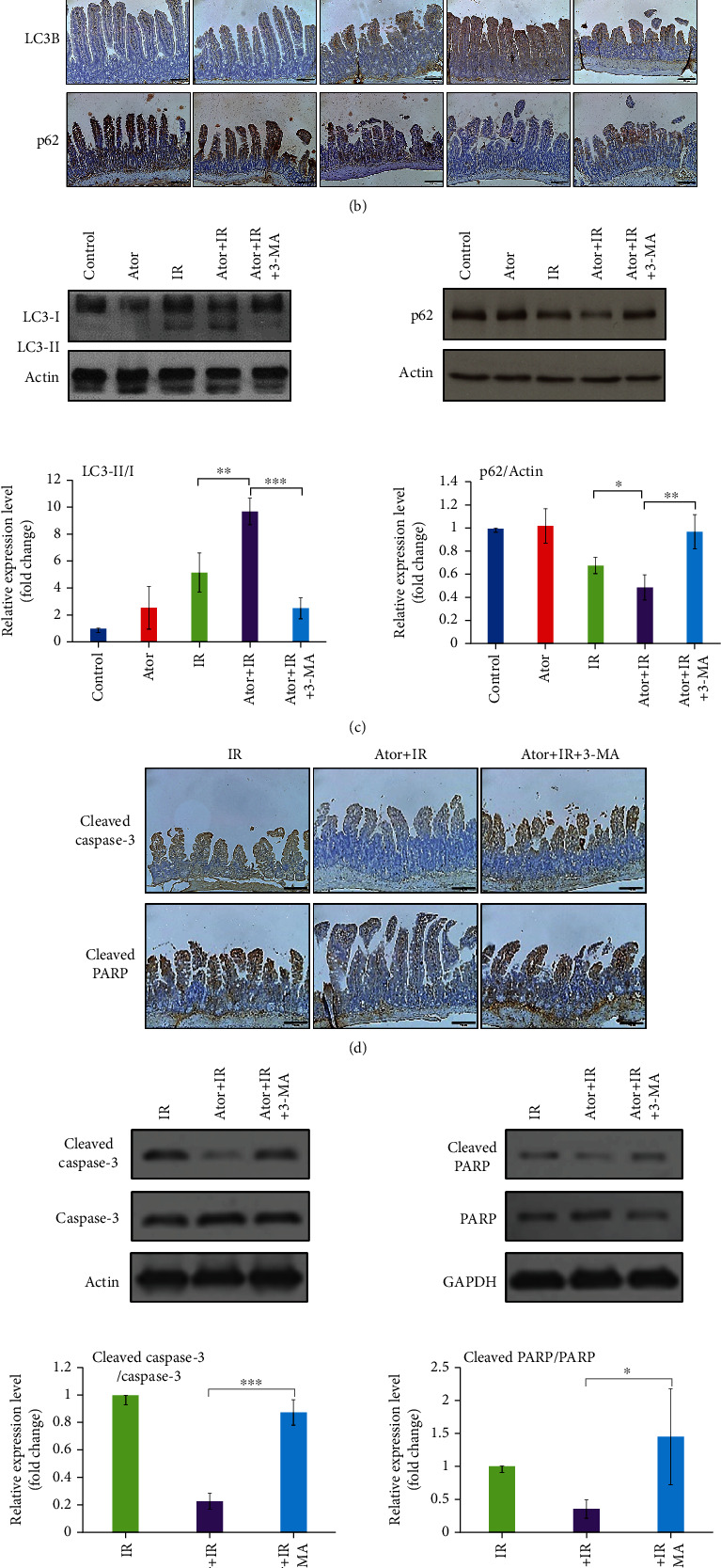
Atorvastatin attenuates RT-induced injury and apoptosis of intestinal epithelium by activating autophagy. (a) Representative H&E images of jejunum tissue sections from five different groups. Scale bar = 100 *μ*m. (b) IHC analysis of LC3B and p62 in jejunum tissue sections. Scale bar = 100 *μ*m. (c) Protein expression analysis for LC3 and p62 by western blot. The relative amount of LC3-II was quantified as the LC3-II to LC3-I ratio, and p62 was quantified as the p62 to actin ratio. The relative ratios of the five groups are represented in the bar graph (the control group is arbitrarily presented as 1). The data are presented as mean ± SE of three independent experiments. ^∗^*P* < 0.05; ^∗∗^*P* < 0.01; ^∗∗∗^*P* < 0.001. (d) IHC analysis was used to compare cleaved caspase-3 and cleaved PARP levels among the IR, Ator+IR, and Ator+IR+3-MA groups. Scale bar = 100 *μ*m. (e) The expression levels of cleaved caspase-3 and cleaved PARP were analyzed by western blot analysis. The bar graph represents the relative ratios of the cleaved protein to the noncleaved protein. The data are presented as mean ± SE of three independent experiments. The relative ratio of the control group is arbitrarily presented as 1. ^∗^*P* < 0.05; ^∗∗∗^*P* < 0.001.

We wanted to examine whether the activation of autophagy, following atorvastatin administration, could protect the intestinal tissue from RT-induced injury. For this purpose, an autophagy inhibitor, 3-MA, was injected intraperitoneally for five days, concurrently with atorvastatin (Supplementary Figure 1). Representative H&E staining images are shown in Figure 2(a). When compared with the scores of the Ator+IR group (modified Chiu's injury score, 0.38 ± 0.52, and V/C ratio, 2.52 ± 0.89), we found that in the Ator+IR+3-MA group, the modified Chiu's injury score was higher (4.13 ± 0.83) and the V/C ratio was lower (1.17 ± 0.39) (Figure 1(b) and Table 1). This suggests that autophagy triggered by atorvastatin administration—prior to RT—is a crucial protective mechanism against RT-induced intestinal injury.

Subsequently, we examined two autophagy-associated molecules. One is the active marker LC3B, which is the most component of autophagosomes, and the other is the inactive marker sequestosome 1 (or p62); both could potentially be altered after the administration of atorvastatin [27]. Based on the IHC staining (Figure 2(b)), we found that the Ator+IR group expressed higher levels of LC3B and lower levels of p62 than the IR group. However, the addition of 3-MA inhibited the expression of LC3B in the mucosa of the Ator+IR+3-MA group compared to that in the Ator+IR group (Figure 2(b)). The p62 expression levels in the IR and Ator+IR+3-MA groups were higher than those in the Ator+IR group (Figure 2(b)). The LC3-II/LC3-I ratio is used to determine the level of autophagy activation. As shown in Figure 2(c), we found that the LC3-II/LC3-I ratio was higher in the Ator+IR group than in the IR group, and this ratio decreased after the administration of 3-MA. The protein expression level of p62, detected by western blotting, showed similar results with lower levels of p62 in the Ator+IR group, as indicated by IHC staining (Figure 2(b)).

We further evaluated the expression levels of cleaved caspase-3 and cleaved PARP in the Ator+IR+3-MA and the Ator+IR groups. As shown in Figure 2(d), we demonstrated that atorvastatin markedly downregulated the expression of both these proteins after RT induction. The administration of 3-MA reversed this effect. Similar results were observed in western blot analysis (Figure 2(e)). This implies that RT-induced apoptosis and damage in the intestinal epithelium can be reduced via autophagy activation stimulated by atorvastatin.

### 3.3. Atorvastatin Attenuates RT-Induced OS and Increases the Expression of Antioxidants

**Figure 3:**
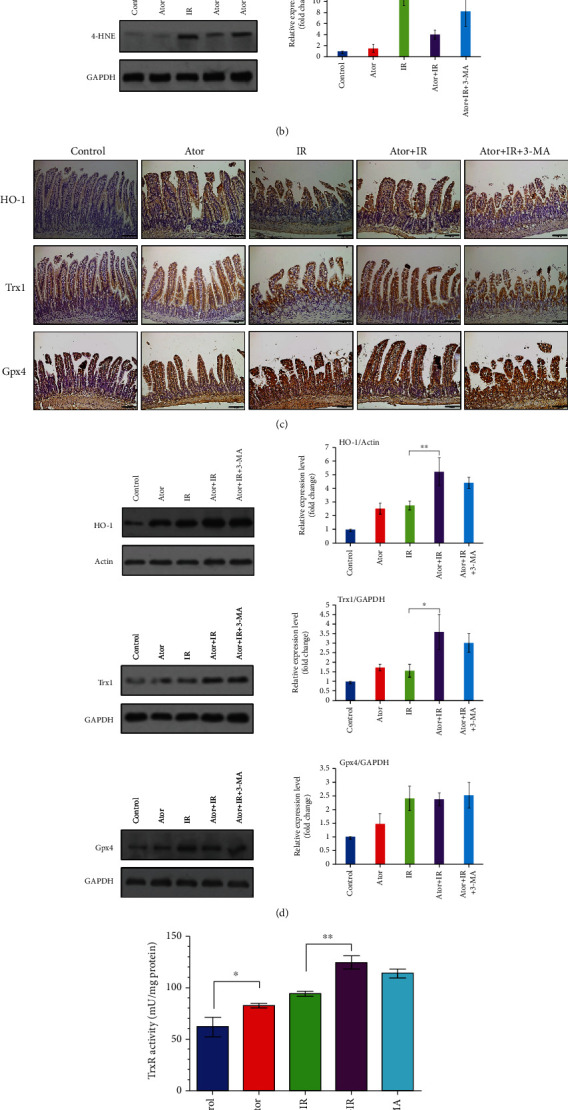
Atorvastatin reduces RT-induced ROS levels by increasing the expression of antioxidants. (a) IHC analysis of the 4-HNE expression in jejunum tissue sections from five groups. Scale bar = 100 *μ*m. (b) The expression level of 4-HNE was determined by western blot analysis. The relative amount of 4-HNE was quantified as the 4-HNE to GAPDH ratio. The relative ratios of the five groups are represented in the bar graph (the control group is arbitrarily presented as 1). The data are expressed as mean ± SE of three independent experiments. ^∗^*P* < 0.05; ^∗∗^*P* < 0.01. (c) IHC analysis of the expression of HO-1, Trx1, and Gpx4 in jejunum tissue sections from five groups. Scale bar = 100 *μ*m. (d) The expression levels of HO-1, Trx1, and Gpx4 were determined by western blot analysis. The relative amounts were quantified as the HO-1, Trx1, or Gpx4 to actin or GAPDH ratios and are represented in the bar graph. The data are expressed as mean ± SE of three independent experiments. The relative ratio of the control group is arbitrarily presented as 1. ^∗^*P* < 0.05; ^∗∗^*P* < 0.01. (e) TrxR activity of five groups was measured and is represented as mU/mg protein. The data are expressed as mean ± SE of three independent experiments. ^∗^*P* < 0.05; ^∗∗^*P* < 0.01.

Statin drugs and the associated autophagy activities have been shown to possess antioxidant effects [28]. As shown in Figures 3(a) and 3(b), IHC staining and western blot analyses revealed that atorvastatin significantly suppressed the level of 4-HNE, a biomarker for reactive OS (ROS) and a highly reactive lipid peroxidation product, after RT induction [29]. In contrast, the addition of 3-MA reversed the effect of atorvastatin in decreasing OS.

In addition to 4-HNE, we also assessed whether atorvastatin could induce the expression of other antioxidant-related molecules, including HO-1, Trx, and Gpx4 [30–32]. As shown in Figures 3(c) and 3(d), expression levels of HO-1 and Trx1 were upregulated in the Ator+IR group compared with those in the IR group; however, the level of Gpx4 remained unaltered. No differences were observed in the expression levels of HO-1, Trx1, and Gpx4 between the Ator+IR and Ator+IR+3-MA groups. The activity of TrxR was significantly increased in the Ator+IR group compared with that in the IR group (*P* = 0.00608), whereas the levels of TrxR between the Ator+IR and Ator+IR+3-MA groups were not significantly different (*P* = 0.12184) (Figure 3(e)). Thus, these findings indicate that atorvastatin administration significantly increased HO-1 and Trx1 expression, but not that of Gpx4. It is possible that this activation pathway may not be associated with the activation of autophagy.

### 3.4. Autophagy Activation Stimulated by Atorvastatin Resists RT-Induced DNA Damage and Inflammation in the Intestinal Epithelium

**Figure 4:**
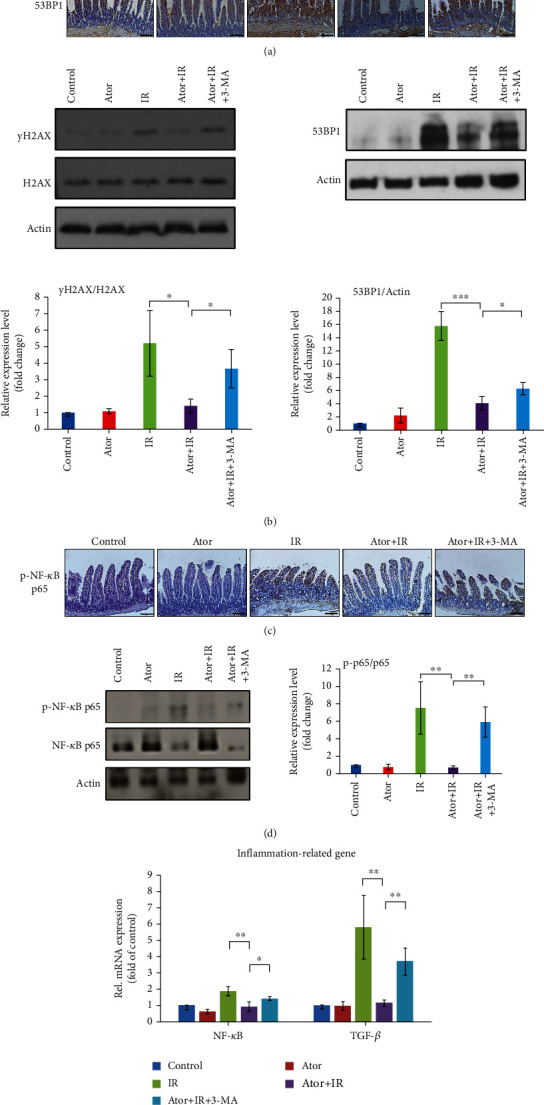
Atorvastatin downregulates DNA damage and RT-induced inflammation. (a) IHC analysis of *γ*H2AX and 53BP1 in jejunum tissue sections from five groups. Scale bar = 100 *μ*m. (b) The expression levels of *γ*H2AX and 53BP1 were analyzed by western blot analysis. The relative amount of *γ*H2AX was quantified as the *γ*H2AX to H2AX ratio and that of 53BP1 was quantified as the 53BP1 to actin ratio. The relative ratios of five groups are represented in the bar graph (the control group is arbitrarily presented as 1). The data are expressed as mean ± SE of three independent experiments. ^∗^*P* < 0.05; ^∗∗∗^*P* < 0.001. (c) IHC analysis of the expression of phospho (p)-NF-*κ*B p65 in jejunum tissue sections from five groups. Scale bar = 100 *μ*m. (d) The relative amount of p-NF-*κ*B p65 was quantified as the p-NF-*κ*B p65 to NF-*κ*B p65 ratio based on western blotting and is represented in the bar graph. The data are expressed as mean ± SE of three independent experiments. The relative ratio of the control group is arbitrarily presented as 1. ^∗^*P* < 0.05; ^∗∗^*P* < 0.01. (e) Total RNA was isolated from intestinal tissue of mice in five groups, and inflammation-related mRNA expressions of NF-*κ*B and TGF-*β* were analyzed by qRT-PCR. ^∗^*P* < 0.05; ^∗∗^*P* < 0.01.

Considering that autophagy maintains genomic stability by alleviating DNA damage [33], we assessed whether atorvastatin administration effectively reduced RT-induced DNA damage by enhancing autophagy levels. IHC analysis revealed that the expression level of *γ*H2AX was downregulated in the Ator+IR group as compared to that in the IR group. However, the level of *γ*H2AX increased in the Ator+IR+3-MA group (Figure 4(a)). Western blot data also revealed similar results for *γ*H2AX (Figure 4(b)). As shown in Figures 4(a) and 4(b), the expression level of 53BP1 was higher in the IR group than in the Ator+IR group, whereas 3-MA inhibited the protective effect of autophagy on DNA damage. Thus, these findings suggest that atorvastatin-induced upregulation of autophagy protects intestinal epithelium against RT-induced DNA damage and subsequent apoptosis.

Statin drugs and autophagy exert anti-inflammatory effects [9, 14]. A previous study showed that atorvastatin inhibited inflammation [34]. Furthermore, it had protective and stabilizing effects on vulnerable atherosclerotic plaques by inducing autophagy [34]. Therefore, we assessed whether atorvastatin could alleviate RT-induced inflammation in the small intestinal epithelium. As shown in Figures 4(c) and 4(d), we observed a lower expression level of p-NF-ĸB p65, mediators of inflammation, in the Ator+IR group than in the IR and Ator+IR+3-MA groups (Figures 4(c) and 4(d)). Additionally, mRNA levels of inflammation-related genes (NF-ĸB and TGF-*β*) were significantly lower in the Ator+IR group compared with those in the IR and Ator+IR+3-MA groups (Figure 4(e)). These results suggested that atorvastatin diminished the degree of inflammation in the small intestine during abdominal RT by activating autophagy.

### 3.5. Atorvastatin Maintains the Intestinal Barrier Integrity after Abdominal RT through Activation of Autophagy

**Figure 5:**
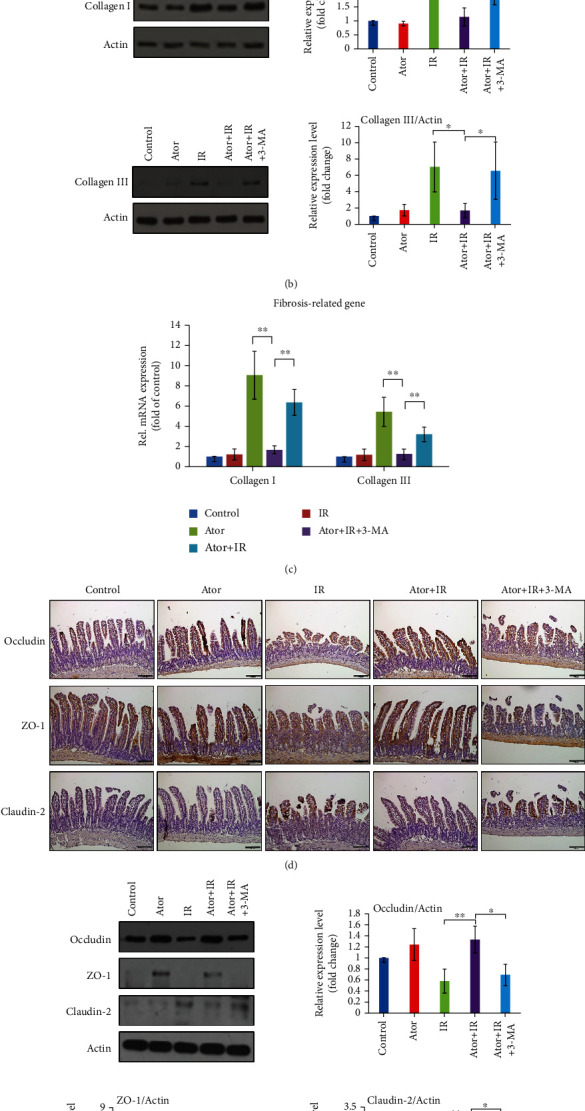
Atorvastatin maintains intestinal epithelium integrity via autophagy activation after RT. (a) IHC analysis of collagen I and III expression in jejunum tissue sections from different groups. Scale bar = 100 *μ*m. (b) The expression levels of collagen I and III were determined by western blot analysis. The relative amount was quantified as collagen I and collagen III to actin ratios and is represented in the bar graph. The data are expressed as mean ± SE of three independent experiments. The relative ratio of the control group is arbitrarily presented as 1. ^∗^*P* < 0.05; ^∗∗∗^*P* < 0.001. (c) mRNA levels of collagen I and III were analyzed by qRT-PCR. ^∗∗^*P* < 0.01. (d) IHC analysis of occludin, ZO-1, and claudin-2 expression in jejunum tissue sections from five groups. Scale bar = 100 *μ*m. (e) The expression levels of occludin, ZO-1, and claudin-2 were determined by western blot analysis. The relative amount was quantified as occludin, ZO-1, and claudin-2 to actin ratios and is represented in the bar graph. The data are expressed as mean ± SE of three independent experiments. The relative ratio of the control group is arbitrarily presented as 1. ^∗^*P* < 0.05; ^∗∗^*P* < 0.01.

We further investigated whether RT-induced destruction of the intestinal barrier, which appears as fibrosis and disruption of tight junctions, could be ameliorated by atorvastatin administration. A previous study determined that statins could attenuate fibrosis in the treatment of muscular dystrophy by autophagy activation [35]. Our IHC and western blot analyses revealed that collagen I and collagen III expressions were reduced in the Ator+IR group compared with that in the IR and Ator+IR+3-MA groups (Figures 5(a) and 5(b)). We found that mRNA levels of collagen I and collagen III were significantly downregulated in the Ator+IR group compared to those in the IR and Ator+IR+3-MA groups (Figure 5(c)). These results suggest that autophagy activated by atorvastatin significantly reduced fibrosis levels in the small intestine, after abdominal RT.

The intercellular adhesion complexes are composed of tight junction proteins between epithelial cells and are responsible for the paracellular permeability of the intestinal barrier [36]. We assessed whether tight junction proteins could be affected by atorvastatin and RT. Therefore, we evaluated occludin and ZO-1; these proteins are responsible for maintaining the integrity of the intestinal epithelium. Furthermore, we examined claudin-2 as well, which is responsible for the impaired barrier of the intestinal epithelium [37, 38]. Our results showed that occludin and ZO-1 expression levels were upregulated in the Ator+IR group compared with those in the IR and Ator+IR+3-MA groups (Figures 5(d) and 5(e)). In contrast, the expression level of claudin-2 was reduced in the Ator+IR group than those in the IR and Ator+IR+3-MA groups (Figures 5(d) and 5(e)). These results indicate that atorvastatin administration maintains the integrity of the intestinal epithelium after RT via autophagy activation.

### 3.6. Atorvastatin-Induced Autophagy Can Reduce the Loss of Intestinal Barrier Integrity and Function after RT

**Figure 6:**
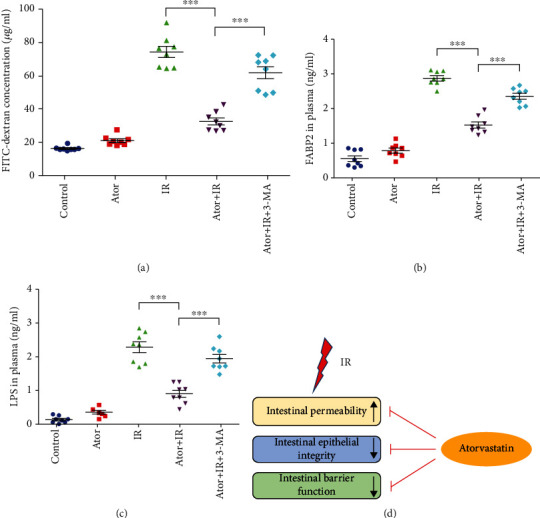
Autophagy activation by atorvastatin alleviates RT-induced loss of mouse intestinal integrity and barrier function. (a) Intestinal permeability was evaluated based on serum FITC-dextran levels of mice from five groups. The concentrations of FITC-dextran are shown in the scatter dot plots. Mean ± SE (*n* = 8) is denoted. ^∗∗∗^*P* < 0.001. (b) Intestinal epithelial integrity was determined by FABP2 ELISA in the plasma. The scatter dot plots show the distribution of the amount of FABP2. Mean ± SE (*n* = 8) is denoted. ^∗∗∗^*P* < 0.001. (c) Intestinal barrier function was assessed by LPS ELISA in plasma. The concentrations of LPS are shown in the scatter dot plots. Mean ± SE (*n* = 8) is denoted. ^∗∗∗^*P* < 0.001. (d) Graphical summary illustrates that the activation of autophagy by atorvastatin reduces RT-induced loss of mice's intestinal epithelial integrity and barrier function.

Damage to the intestinal epithelial barrier often leads to the loss of absorption and metabolic function. Therefore, we further assessed whether atorvastatin administration can reduce RT-induced disruption of intestinal physiological functions, including intestinal epithelium barrier permeability and integrity. As shown in Figure 6(a), intestinal barrier permeability was determined by measuring the level of serum FITC-dextran that permeated across the intestinal lumen into the blood. Compared to the levels in the IR and Ator+IR+3-MA groups, the level of FITC-dextran in the Ator+IR group was significantly decreased (Figure 6(a)).

Previous studies have shown that plasma FABP2 and plasma LPS are markers for loss of intestinal epithelial barrier and dysfunction, respectively [39, 40]. We found that the levels of plasma FABP2 and LPS were significantly lower in the Ator+IR group than those in the IR and Ator+IR+3-MA groups (Figures 6(b) and 6(c)). These findings indicate that, after RT, through the activation of autophagy, atorvastatin could reverse the increased barrier permeability and disruption of barrier integrity and function of the intestinal epithelium (Figure 6(d)).

## 4. Discussion

In this study, we used jejunum to perform our assays, as it is a part of the small intestine where significant absorption occurs. Damage to the jejunum mucosa may be reflected via malabsorption and malnutrition. We found that atorvastatin attenuated the RT-induced increase in modified Chiu's injury score through antiapoptosis, anti-DNA damage, anti-OS, anti-inflammation, and antifibrotic effects and protected the integrity and function of the intestinal barrier. However, these biological effects were impeded on administering an autophagy inhibitor, thus indicating that the mechanisms by which intestinal injury is reduced by atorvastatin are partially mediated by activating autophagy. Additionally, we discovered that atorvastatin administration exerted an antioxidant effect when the intestinal epithelium was subjected to RT, a phenomenon independent of the autophagy activation.

Patients who receive abdominal and pelvic RT can experience acute or chronic enteritis [41]. This can limit escalating RT doses and substantially attenuate the effectiveness of RT in cancer cells. Previous studies have demonstrated that statins exert radioprotective effects in normal tissues by attenuating RT-induced damage [42–44]. Our current study showed that atorvastatin treatment attenuated RT-induced intestinal epithelium damage, as indicated by the low modified Chiu's injury scores and an increased V/C ratio. The mechanisms of atorvastatin that decreased RT-induced intestinal injury mainly involved the downregulation of apoptosis-related molecules, such as cleaved caspase-3 and cleaved PARP. RT can also cause DNA damage in normal and cancer cells by activating the apoptotic pathway [45]. We found that atorvastatin led to downregulation of the DNA damage-related molecules, namely, *γ*H2AX and 53BP1, in intestinal cells post-RT. These findings are in line with a study by Ziegler et al., which states that administration of statins could decrease the DNA damage response induced by anticancer therapy in normal tissue cells [46].

RT also induces an inflammatory response and fibrosis in normal cells by producing chemokines and proinflammatory cytokines, such as TNF-*α*, IL-1*α*, IL-6, TGF-*β*, and NF-*κ*B [47, 48]. TGF-*β* further promotes the proliferation of collagen-producing myofibroblasts in response to RT [49]. Myofibroblasts are crucial effector cells that promote organ fibrosis by producing many extracellular components, including collagen I, collagen III, and glycoproteins [50]. Previous studies have shown that Trx1 can decrease OS by activating the production of HO-1 [51, 52]. Our results showed that atorvastatin treatment downregulated expression levels of ROS (4-HNE), inflammation markers (p-NF-*κ*B p65 and TGF-*β*) and fibrosis markers (collagen I and collagen III) in intestinal cells that were triggered by RT. This indicates that atorvastatin can prevent RT-induced intestinal injury by attenuating these factors. Additionally, we found that atorvastatin administration upregulated the expression of HO-1 and Trx and the activated status of TrxR stimulated by RT compared with RT alone. To our knowledge, our study is the first to demonstrate that atorvastatin can efficiently protect intestinal epithelial cells against RT-induced OS and exert antioxidant effects *in vivo*.

Intestinal epithelial cells are connected by two main junction complexes. One of them consists of occludin, ZOs, claudin, and junctional adhesion protein molecule-A, and the other adherence junction is composed of *α*-catenin, *β*-catenin, and E-cadherin [53]. Previous studies revealed that knockdown of occludin leads to impairment of intestinal epithelium barrier function [54]. Furthermore, the upregulation of occludin and ZO-1 triggered by ginseng-derived small molecule oligopeptides may be linked to the reduction of RT-induced permeability of the intestinal epithelium [54]. As opposed to occludin and ZO-1, the high expression of claudin-2 has been associated with the severity of inflammatory diseases of the gut [55, 56]. Using transgenic mouse models, Raju et al. revealed that deficiency of claudin-2 diminished the junction barrier leakage and delayed the progression of colitis [57]. Our current study showed that administration of atorvastatin decreased intestinal damage resulting from RT and improved intestinal integrity by upregulating occludin and ZO-1 expression and downregulating claudin-2 expression. In addition, we observed that atorvastatin significantly decreased the concentration of serum FITC-dextran (increased barrier permeability), plasma FABP2 (disruption of barrier integrity), and LPS (loss of barrier function) when compared with the corresponding levels in the RT group.

Autophagy is an important process that exerts radioprotective effects in normal tissue cells during RT [58, 59]. It has been proven that atorvastatin activates autophagy [18–20]. Statins can also alleviate inflammation and fibrosis in cells by upregulating autophagy [34]. These findings are consistent with our current findings that atorvastatin upregulates autophagy-related signals in the intestinal epithelium. Our findings further demonstrated that the inhibitory effects, induced by atorvastatin, on apoptosis, DNA damage, inflammation, fibrosis, intestinal permeability, and intestinal barrier disintegrity and dysfunction, were reversed after the administration of an autophagy inhibitor (3-MA).

**Figure 7:**
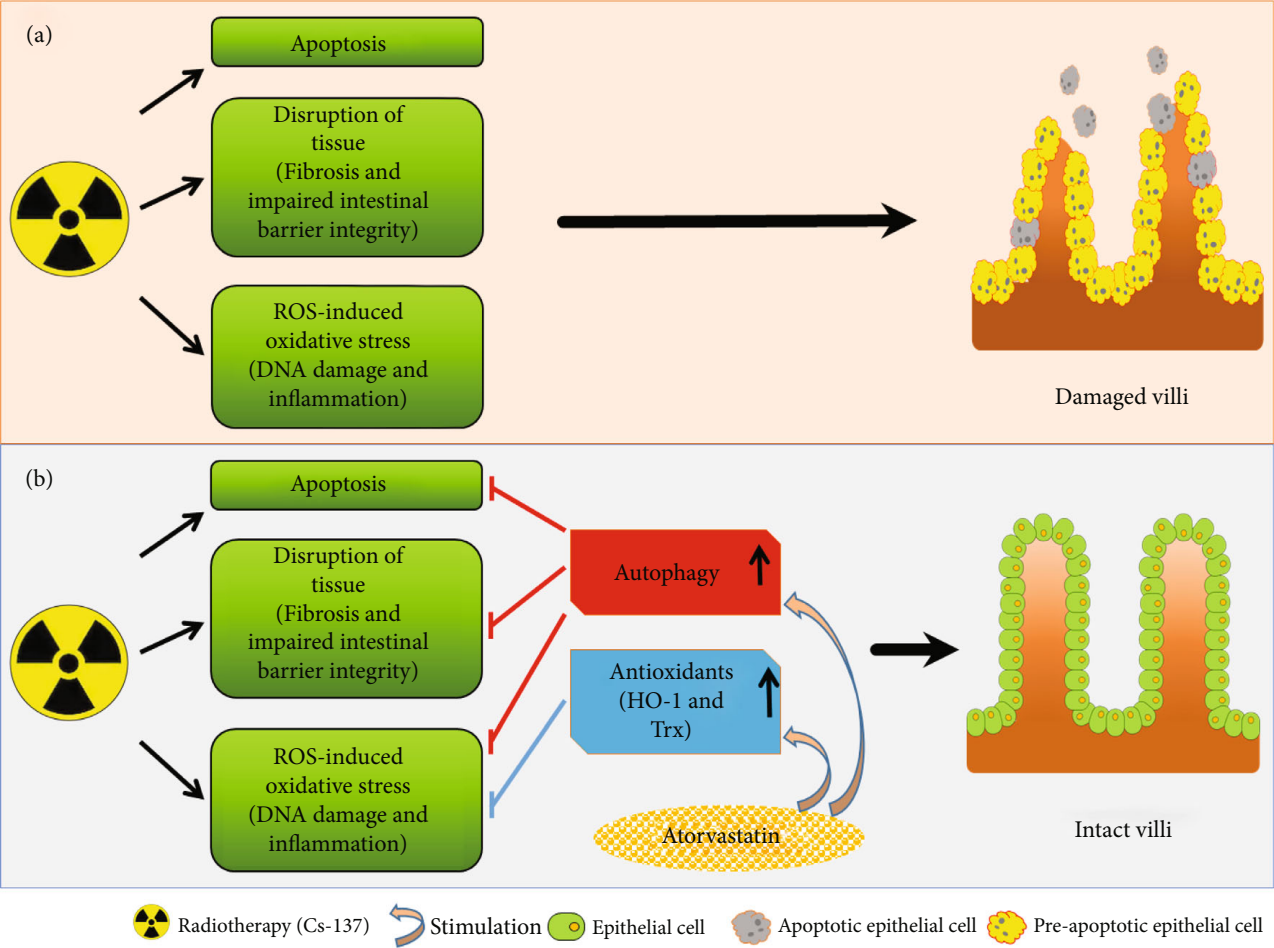
Radioprotective capability of atorvastatin in the intestinal epithelium. (a) RT resulted in apoptosis, ROS-induced oxidative stress (DNA damage and inflammation), and the impairment of intestinal epithelium (fibrosis and impaired intestinal barrier integrity) in our murine model. (b) The expression level of autophagy-associated molecules might determine the integrity of the intestinal tissue after RT. Atorvastatin administration activates the biological functions of autophagy, including attenuating apoptosis, DNA damage, oxidative stress, inflammation, and fibrosis, and thus decreases the injury score of the intestinal epithelium. Additionally, atorvastatin administration stimulates the production of antioxidants molecules, such as HO-1 and Trx. Therefore, atorvastatin may effectively help escalate RT dose to increase tumor control in patients receiving abdominal and pelvic RT by reducing RT-associated enteritis.

However, the antioxidant stress activity of atorvastatin, through HO-1 and Trx-1, to protect the intestinal epithelium against post-RT injury was independent of the autophagy activation. Thus, atorvastatin exerted protective effects via antiapoptosis effect, decreasing DNA damage, anti-inflammatory effect, diminishment of the intestinal epithelial permeability, maintenance of barrier integrity and function, and anti-ROS effect. Furthermore, protective effects were exerted by also activating antioxidant molecules, namely, HO-1 and Trx-1 (Figure 7).

Most radioprotective compounds can diminish normal tissue injury without limiting their antitumor efficacies [60]. Previous studies have shown that statins inhibit the growth of tumor cells by downregulating PTEN/AKT pathway signals, thus enhancing antiangiogenesis and stimulating immunomodulatory effects [61–63]. The use of statin is closely associated with a higher survival in patients with resectable colorectal cancer [64]. Improved overall survival rate of patients with hepatocellular carcinoma who received palliative treatment [65], increased progression-free survival of high-grade endometrial cancer patients [66], and improved freedom from biochemical failure in patients with prostate cancer [67] were observed.

The current study did not compare certain important biological functions between mice groups that received atorvastatin concurrently with RT and those groups that received atorvastatin before RT. The following biological functions could be potentially evaluated: the reduction of RT-induced intestinal mucosa injury, maintenance of intestinal barrier integrity, and function of atorvastatin. We expect that patients who are administered atorvastatin before RT, during RT, and after RT will be protected against RT-induced enteritis. Our preclinical findings provide translational evidence that patients who are continuously administered atorvastatin, while receiving abdominal or pelvic RT, would be protected against the RT-associated small intestinal toxicities.

## 5. Conclusion

In conclusion, our results indicated that atorvastatin administration could decrease RT-induced intestinal epithelium damage, inflammation, and fibrosis. The intestinal integrity and barrier function were maintained by activating autophagy-regulated biological functions. Additionally, our results demonstrated that atorvastatin administration exerted antioxidant effects, thus diminishing the impairment of the intestinal epithelium caused by RT-induced OS. These preclinical findings may allow clinicians to escalate RT doses to concomitantly treat abdominal or pelvic cancer patients while administering atorvastatin so as to reduce the degree of RT-associated enteritis. Further studies are warranted to investigate whether the administration of atorvastatin could synergistically enhance the antitumor effects of RT in intestinal cancers.

## Figures and Tables

**Table 1:** Value of villus/crypt (V/C) ratio in each group.

Groups.	Sites		
	Villi height (*μ*m)	Crypt death (*μ*m)	V/C ratio
Control	300.61 ± 56.38	94.02 ± 21.57	3.34 ± 0.96
Ator	269.27 ± 100.87	97.77 ± 25.56	2.84 ± 1.04
IR	134.82 ± 29.08	99.26 ± 20.18	1.41 ± 0.43
Ator+IR	241.55 ± 58.22	103.06 ± 26.94	2.52 ± 0.89
Ator+IR+3-MA	125.40 ± 41.74	111.67 ± 32.39	1.17 ± 0.39

Data are presented as *mean* ± *SE* (*n* = 8).

## Data Availability

Data is contained within the article.

## Supplementary Materials

Supplementary 1 Supplementary Figure 1: schematic depiction of the experimental design for the treatment regimen of five *in vivo* experimental groups (control, atorvastatin, irradiation (IR), atorvastatin+IR, and atorvastatin+IR+3-MA). This design was established to determine the radioprotective mechanism induced by atorvastatin and to assess the role of autophagy activated by atorvastatin in the radioprotective mechanism.

Supplementary 2 Supplementary Figure 2: experimental setup for the assessment of RT-induced intestinal tissue damage and the protective effect of atorvastatin in combination with RT. C57BL/6 mice were orally administered atorvastatin for five days, after which the abdomens of mice were irradiated. The dose was set to 5 Gy and was administered for three days using a Cs-137 irradiator. A lead block was used to shield the rest of the body. A day after fasting, the jejunum segment was excised for further analyses.

Supplementary 3 Supplementary Table 1: blood biochemistry analysis and hematology assays from four treated groups.
