# Supplementary material for: Atorvastatin Attenuates Radiotherapy-Induced Intestinal Damage through Activation of Autophagy and Antioxidant Effects
Source: Oxid Med Cell Longev. 2022 Aug 31;2022:7957255. doi: 10.1155/2022/7957255 (PMC9459441; doi:10.1155/2022/7957255)
Supplement: Supplementary 1 — Supplementary Figure 1: schematic depiction of the experimental design for the treatment regimen of five in vivo experimental groups (control, atorvastatin, irradiation (IR), atorvastatin+IR, and atorvastatin+IR+3-MA). This design was established to determine the radioprotective mechanism induced by atorvastatin and to assess the role of autophagy activated by atorvastatin in the radioprotective mechanism. [file 7957255.f1.pdf]

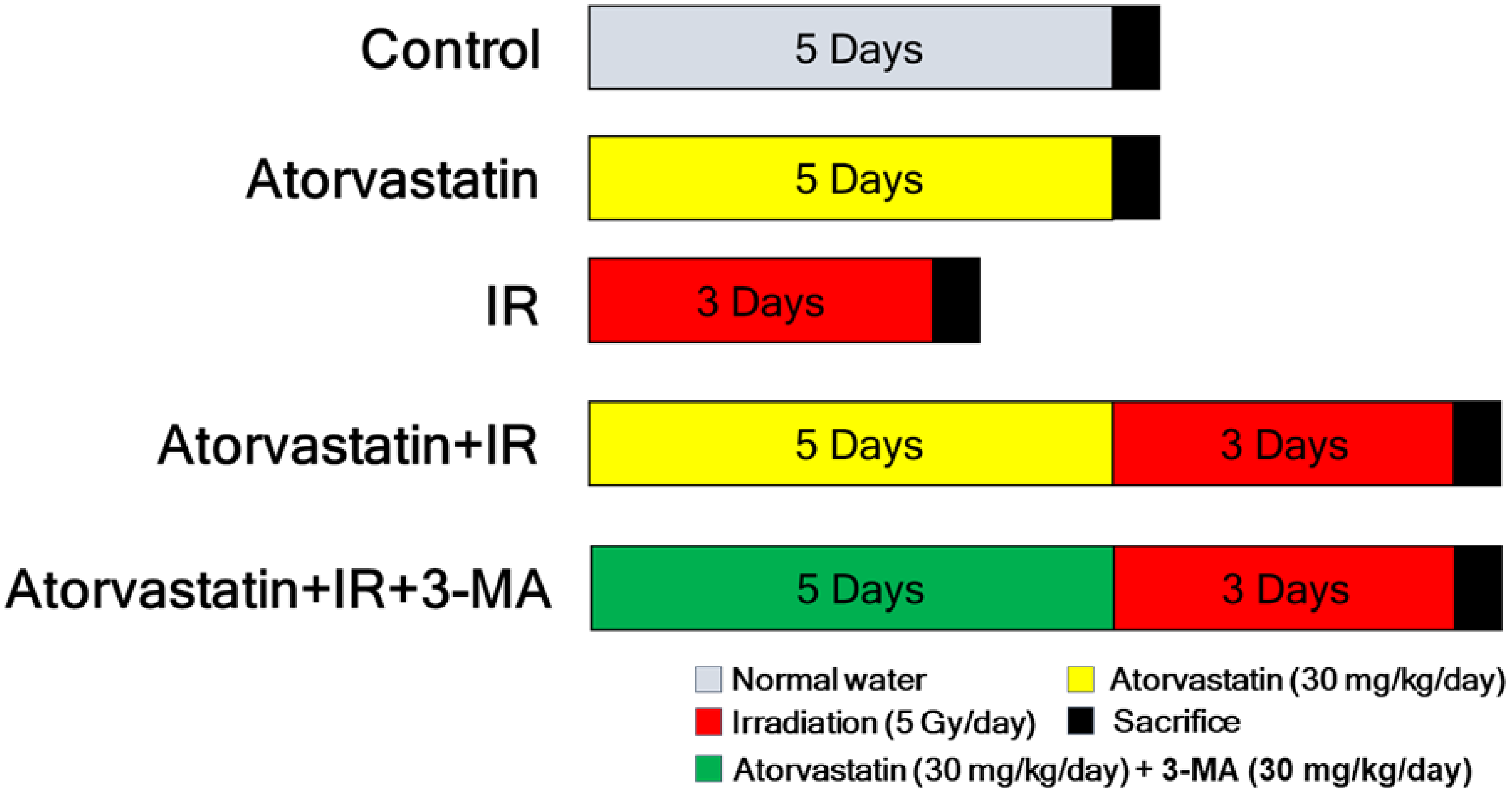

Supplementary Figure 1. Schematic depiction of the experimental design for the treatment regimen of five *in vivo* experimental groups (control, atorvastatin, irradiation [IR], atorvastatin+IR, and atorvastatin+IR+3-MA). This design was established to determine the radioprotective mechanism induced by atorvastatin and to assess the role of autophagy activated by atorvastatin in the radioprotective mechanism.
