# Supplementary material for: Atorvastatin Attenuates Radiotherapy-Induced Intestinal Damage through Activation of Autophagy and Antioxidant Effects
Source: Oxid Med Cell Longev. 2022 Aug 31;2022:7957255. doi: 10.1155/2022/7957255 (PMC9459441; doi:10.1155/2022/7957255)
Supplement: Supplementary 2 — Supplementary Figure 2: experimental setup for the assessment of RT-induced intestinal tissue damage and the protective effect of atorvastatin in combination with RT. C57BL/6 mice were orally administered atorvastatin for five days, after which the abdomens of mice were irradiated. The dose was set to 5 Gy and was administered for three days using a Cs-137 irradiator. A lead block was used to shield the rest of the body. A day after fasting, the jejunum segment was excised for further analyses. [file 7957255.f2.pdf]

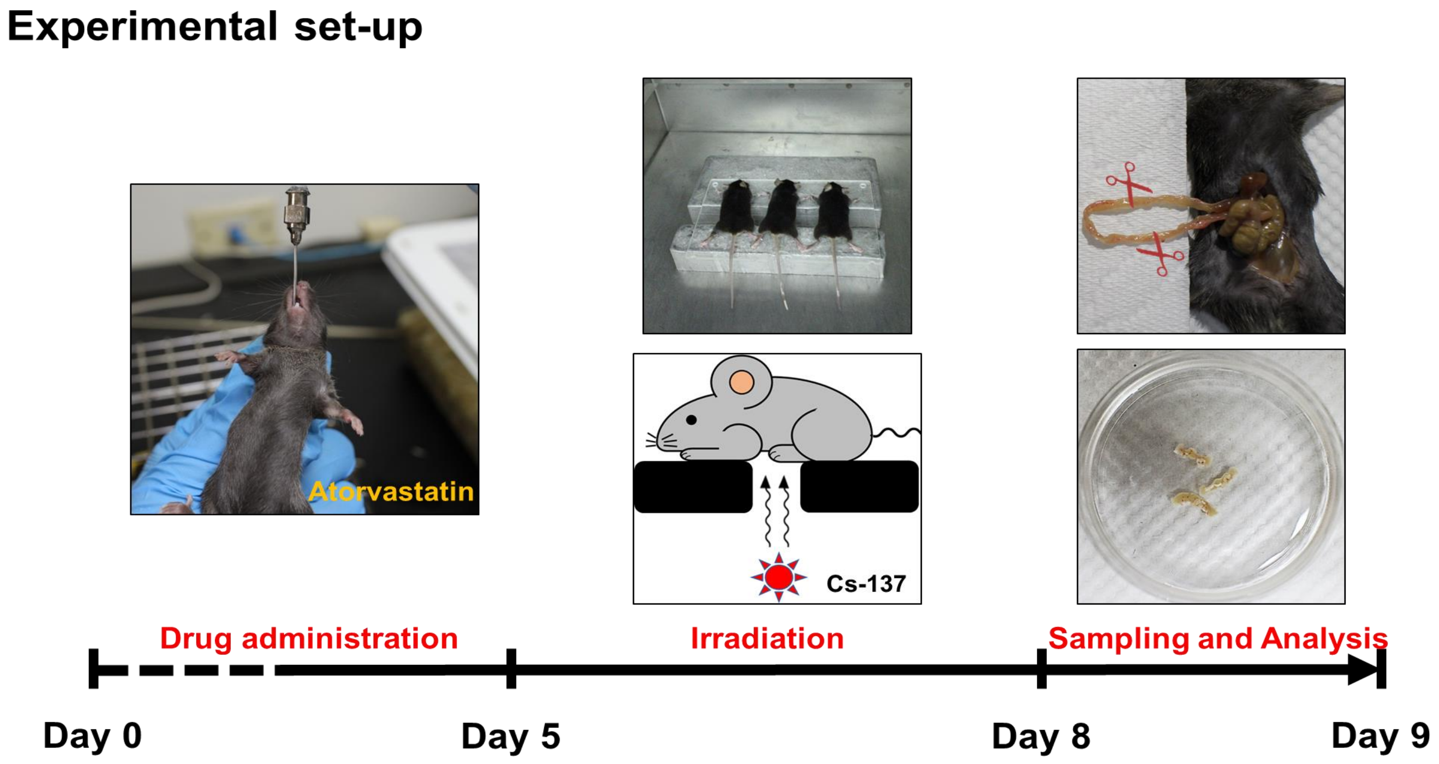

Supplementary Figure 2. Experimental setup for the assessment of RT-induced intestinal tissue damage and the protective effect of atorvastatin in combination with RT. C57BL/6 mice were orally administered atorvastatin for five days, after which the abdomens of mice were irradiated. The dose was set to 5 Gy and was administered for three days using a Cs-137 irradiator. A lead block was used to shield the rest of the body. A day after fasting, the jejunum segment was excised for further analyses.
