# Supplementary material for: Atorvastatin Attenuates Radiotherapy-Induced Intestinal Damage through Activation of Autophagy and Antioxidant Effects
Source: Oxid Med Cell Longev. 2022 Aug 31;2022:7957255. doi: 10.1155/2022/7957255 (PMC9459441; doi:10.1155/2022/7957255)
Supplement: Supplementary 3 — Supplementary Table 1: blood biochemistry analysis and hematology assays from four treated groups. [file 7957255.f3.pdf]

Supplementary Table 1: Blood biochemistry analysis and hematology assays from four treated groups.

Parameter	Normal		IR	
	-	Atorvastatin	-	Atorvastatin
**Biochemistry**				
ALB (g/L)	35.8±1.91	35.78±1.8	32.43±2.72	30.9±1.12
ALT (U/L)	18±1.41	19±4.4	16.25±3.1	14.6±2.07
CREA (mg/dL)	0.22±0.043	0.21±0.035	0.16±0.039	0.15±0.034
LDH (U/L)	840.75±8.92	807.25±76.45	1530±336.23	1160.75±214.07
**Hematology**				
HGB (g/dL)	13.65±0.25	15.12±1.15	15.13±0.56	14.2±0.99
WBC (K/uL)	6.99±0.54	8.13±1.59	0.26±0.19	0.34±0.04
PLT (K/uL)	683.25±105.66	869±181.48	939.75±132.46	898±82

Data are presented as mean ± standard error (n = 8)
